# A Set of Multiresistant Isolates of *Mycoplasma bovis* Subtype ST-1 with a Variable Susceptibility to Quinolones Are Also Circulating in Spain

**DOI:** 10.3390/pathogens13040329

**Published:** 2024-04-16

**Authors:** Juan Carlos Corrales, Antonio Sánchez, Xóchitl Hernández, Joaquín Amores-Iniesta, Antón Esnal, Christian de la Fe

**Affiliations:** 1 Ruminant Health Research Group, Department of Animal Health, Faculty of Veterinary Sciences, University of Murcia, 30100 Murcia, Spain; jcorral@um.es (J.C.C.); asanlope@um.es (A.S.); xochitl.hernandez@um.es (X.H.); jamores@um.es (J.A.-I.); 2 Analítica Veterinaria, 48100 Mungía, Vasque Country, Spain; antonesnal@analiticaveterinaria.com

**Keywords:** *Mycoplasma bovis*, biotype ST-1, antimicrobial resistance, QRDR, Spain

## Abstract

*Mycoplasma bovis* (*M. bovis*) is one of the worldwide most important infectious agents involved in respiratory complex diseases (RCD). In Spain, the endemic presence of subtypes ST-2 and ST-3 with phenotypic differences linked to their susceptibility to fluoroquinolones opened the way to develop control strategies focused on previous diagnosis of the subtype and the use of directed therapies when *M. bovis* were involved in RCD. Surprisingly, microbiological studies conducted during 2023 evidenced for the first time the presence of Spanish isolates of a new *polC*-subtype, previously classified as ST-1, recovered from calves with respiratory symptoms and pneumonia in different areas of the country (*n* = 16). Curiously, the minimum inhibitory concentration (MIC) to a panel of antimicrobials revealed phenotypic differences between these ST-1 isolates when using fluoroquinolones (FLQ). There is no geographical correlation between MIC profiles even for a set of 8 isolates recovered from different animals in the same flock. Sequencing of 4 genes (*gyrA*, *gyrB*, *parC* and *parE*) encoding quinolone resistance-determining regions (QRDR) evidenced the presence of accumulate mutations in 2 ST-1 isolates with high FLQ MICs, but not in all them (*n* = 3), thus suggesting that, as previously recorded for ST-2 isolates, other mechanisms should be involved in the acquisition of resistence to these antimicrobials. Additionally, as previously detected in the Spanish ST-2 and ST-3, subtype ST-1 isolates are also resistant to macrolides or lincosamides.

## 1. Introduction

*Mycoplasma bovis* is one of the most important worldwide pathogens involved in the respiratory complex diseases (RCD), but also implied in the presence of clinical or subclinical mastitis in dairy cows. Moreover, this bacterium is associated with a high variety of clinical manifestations, including pneumonia, mastitis but also arthritis, keratoconjunctivitis, otitis media or genital disorders [1,2,3]. The infection is highly disseminated throughout Europe, with multiple reports of the presence of *Mycoplasma bovis* [1,2,3,4]. In this sense, and despite the importance of the bovine sector in Spain, where many animals arrive for intensive fattening in different areas, the existing information regarding the presence and importance of *Mycoplasma bovis* is relatively recent. Several studies carried out in the period 2017–2021 showed the extent of *Mycoplasma bovis* infection in the country, both in dairy herds and mainly in calf feedlots. The presence of animals infected by *Mycoplasma bovis* could be detected independently of the presence or not of symptoms, in a similar proportion, thus demonstrating the enormous complications of controlling this infection in an industry where the movement and mixing of animals is frequent and continuous in the fattening groups [5,6]. Until this period, information available was limited to the use of a limited number of Spanish isolates in studies of variability or susceptibility to antimicrobials [7,8,9].

Within feedlots, the infection is endemic, an epidemiological picture is continuously complicated by the entry of new strains [5,6,7]. This leads to a continuous transmission of the agent, mainly excreted via the respiratory route, favored by several factors such as the stress of transport or the mixing of individuals from different origins [10]. The emergence of affected animals is a matter of time, not depending on aspects such as molecular characteristics or even sensitivity to antimicrobials [7]. In the same line, many animals that do not respond to antibiotics are finally sacrificed [5,6,7].

The use of valid and rapid techniques to characterize the microorganisms is essential to study their evolution and degree of variability. Different techniques such as multilocus sequence typing (MLST) or whole genome sequencing (WGS) have been developed in the last years, opening the way to an in-depth study the molecular differences between the strains. Despite that fact, its direct application for controlling the infection on farm results complex, because of cost and time required to have the results. In this sense, in the case of *Mycoplasma bovis*, the partial sequencing of the *polC* gene proved to be a reliable technique for the analysis and classification of isolates [7]. Until now, the presence of at least 5 biotypes of *M. bovis* strains (ST-1 to ST-5) have been described [11]. This system demonstrated the loss of variability of the microorganism in France or the presence of two biotypes (ST-2 and ST-3) circulating in some countries such as France or Spain, although, curiously, in different proportions, with the ST-2 type predominating in France, unlike in Spain [5,7]. Interestingly, characterization studies also suggested the presence of phenotypic differences among them related to their ability to acquire FLQ resistance in vitro [5,11]. In effect, the molecular studies conducted in Spain on more than 100 *Mycoplasma bovis* isolates showed the presence of two *polC* ST-biotypes (ST-2 and ST-3) circulating in the country, with phenotypic differences. While most of the ST-2 strains were sensitive to this group of antimicrobials, most of the ST-3 ones evidenced resistance, thus opening the way to conduct directed treatments based on the epidemiological characteristics of the isolates [5].

All these data have motivated the interest in establishing fast characterization systems for circulating isolates, based on molecular characterization of the partial sequence of *polC* gene that also allow, if necessary, to guide treatments. But surprisingly, the data obtained throughout 2023 allowed the detection of *Mycoplasma bovis* ST-1 isolates circulating in Spain. Due to the lack of data about this subtype, the present work analyses the epidemiological characteristics of the Spanish isolates of this subtype, its sensitivity to the main groups of antimicrobials, with special emphasis on its susceptibility to fluoroquinolones.

## 2. Materials and Methods

### 2.1. Animals

The isolates analyzed were mostly recovered from calves with respiratory symptoms located in fattening farms in Spain. Epidemiological data available is showed in Table 1.

### 2.2. Presence of Mycoplasmas, Identification and Subtyping

For the isolation of mycoplasmas in the swab samples analyzed, they were incubated for 24 h at 37 °C in 2 mL of the specific SP4 medium [12] with some modifications described in Appendix A. After this time, the samples were filtered through 0.45 μm, using membrane filters (LLG-Labware, Dorchester, UK), and then incubated again under the same conditions for 1 week. After this time, a 10 µL aliquot was inoculated into solid SP4 medium, and the plates were incubated at 37 °C until the presence of typical mycoplasma colonies was detected. After 15 days, the samples were considered negative.

In positive cultures, a DNA extraction procedure was performed from 200 µL of culture [13], to subsequently identify the presence of *M. bovis* by PCR [14]. Following the methodology described for working with mycoplasmas, the positive samples were cloned three times by selection of individual colonies, and the presence of this microorganism was again confirmed by PCR at the end of the procedure.

In order to subtype the *M. bovis* colonies present, the polC gene sequence analysis was performed, following the methodology described in the literature [7]. The sequencing was performed at the Biology Service of the University of Murcia, and the analysis of the sequence obtained was carried out using the MEGA 11 software [15].

### 2.3. MIC Assays with M. bovis Isolates

For the development of MIC assays with *M. bovis* isolates, a number of antimicrobials commonly used against mycoplasmosis were selected, including several macrolides, quinolones, tetracyclines, a pleuromutilin and a lincosamide. The specific listing and range of concentrations used per group is shown below: (i) fluoroquinolones: enrofloxacin, marbofloxacin and danofloxacin tested from 8 µg/mL to 0.0625 µg/mL, (ii) two different tetracyclines: doxycycline and oxytetracycline, tested from 8 µg/mL to 0.12 µg/mL, (iii) the pleuromutilin, valnemulin hydrochloride, tested from 4 µg/mL to 0.03 µg/mL, the (iv) the lincosamide, lincomycin and finally a set of macrolides (v), including tulathromycin gamithromycin, tylosine, spyramycin and erythromycin, both groups tested from 8 µg/mL to 0.12 µg/mL. The antimicrobials were supplied by Thermofisher (Illkirch-Graffenstaden, France).

For the development of the technique, all *M. bovis* strains were cultured in SP4 [12] without antibiotics until the stationary growth phase was reached. Sodium pyruvate (0.5%) and phenol red (0.005%) were added to the formulation. Prior to performing the antimicrobial susceptibility test, all cultures were titrated using the previously described method for mycoplasma species [16]. Finally, MIC was performed in duplicate for each strain, in 96-well microtiter plates, using the microbroth dilution method previously described [17], in which a total of 50 µL of each diluted *M. bovis* inoculum (103–105 CFU/mL) was added to another 150 µL of culture medium, using a positive and a negative control of the technique. Once the plates have been prepared, they are incubated for 48 h at 37 °C before proceeding to analyze the resulting colour change for each strain and antimicrobial. The minimum concentration of antibiotic that the growth of each culture is able to inhibit is the MIC. The combined analysis of all the results is what allows establishing the MIC 50 and MIC 90 respectively, defined as the minimum concentration of antimicrobial that is able to inhibit the growth of 50% and 90% of the strains analyzed respectively. To check the validity of the assays, the results obtained in each of the replicates with each strain and antibiotic should not differ by more than one dilution, using the higher MIC value as definitive. Otherwise, a third MIC test was performed, the final value being the result of averaging the three values obtained with each strain.

## 3. Results

### 3.1. Mycoplasma bovis ST 1 Is Also Circulating in Spain with a Variable Antimicrobial Susceptibility to FLQ

**Figure 1:**
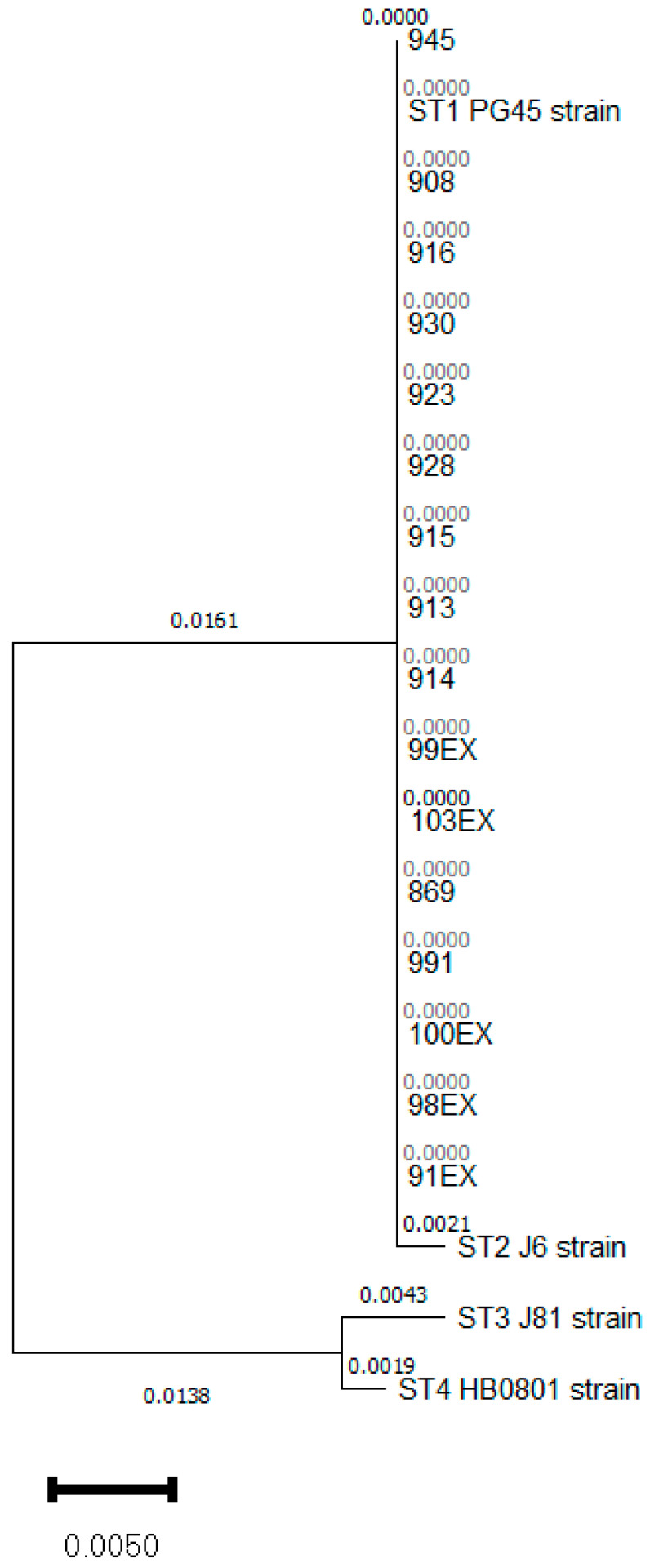
Phylogenetic tree of *polC* gene biotypes analysis. The tree was constructed by using the neighbor-joining method, the Tamura-Nei genetic distance model. Evolutionary analyses was conducted in MEGA X.

Based on the system established to characterize the isolates of *M. bovis* circulating in Spain, a set of 38 *Mycoplasma bovis* strains isolated in 2023 were analyzed using the partial sequencing of the *polC* gene [7]. A total of 16 strains were then classified into the ST-1 biotype (Figure 1). The rest of the bacteria were classified as ST-2 and ST-3. ST-1 isolates were recovered from samples collected in Aragón, Castile La-Mancha, Castile and León, Catalonia or the Region of Murcia, thus representing 5 of the main important livestock areas of the country. All the isolates identified as ST-1 were present in beef cattle with clinical signs of respiratory disease. Moreover, a set of 8 isolates (908, 916, 913, 914, 923, 915, 928 and 930) were recovered from different animals placed at the same feedlot at the Region of Murcia, and thus used to obtain information about the grade of variability of the Spanish strains of this biotype between and inside the herds.

The MIC values obtained for the isolates classified as ST-1 are shown in Table 2. Therefore, most of the *M. bovis* Spanish field isolates have a similar antimicrobial susceptibility profile against macrolides, lincomycin with high MIC values and for valnemulin with low MIC values. Susceptibility profile against tetracyclines seems to be improved despite a set of strains still had high MICs, but its use, especially for oxytetracycline, could be recommended in certain cases. However, a high variability was observed for values for FLQ. Interestingly, significant changes between the ST-1 isolates MIC values for FLQ were observed. Thus, at least 3 groups of ST-1 isolates with different MIC profiles for enrofloxacin were observed: the isolates (869, 923, 928, 930 and 945) (high MIC, even for marbofloxacin and danofloxacin), the isolates 100EX, 103EX, 98EX, 99EX, 91EX, 908, 916, 913, 914 and 915 (low MIC, even for marbofloxacin and danofloxacin), except the isolate 916 that had high MIC value for danofloxacin. Finally, the isolate 991 seems to be an intermedium profile between the other groups.

There is no geographical correlation between MIC profiles even for the isolates recovered at the same time in the same flock. In effect, isolates with at least 3 different MIC profiles were collected at the same day from the 8 beef cattle affected, thus it is unprovable to consider the presence of different evolutions from an initial isolate.

Finally, valnemulin was the only molecule that demonstrated activity against both STs.

### 3.2. Analysis of Point Mutations Conferring Resistance to Quinolones

All the 16 *M. bovis* isolates belonging to ST-1 (*n* = 16) were sequenced to compare nucleotide changes at QRDR genes (Table 3).

Nucleotide changes at QRDR were observed in 2 isolates with the higher MICs for these antimicrobials, mainly located in *gyrB* and especially *parC* in the strain 945. The analysis did not reveal any non-synonymous mutations for ST-1 isolates, but the results should be taken with precaution due to the limited number of strains analyzed. The isolate 869 was characterized by a substitution from serine to phenylalanine at *gyrA* codon 83 (Ser83Phe) and serine to isoleucine at *parE* codon 80 (Ser278Ile). Changes at *gyrB* were also registered (Asp105Asn).

Notably one *M. bovis* isolate (945), demonstrated a high resistance to fluoroquinolones within a unique mutation in the QRDR in the gene *pare* (Gln109Asn). In addition, this isolated showed mutations out of the QRDR but within the *gyrB* gene (Arg58Asn) and *parC* gene (Ser200Glu, Ser230Cys, Asp273Thr, Ala276Leu and Val283Phe-Leu-Ala). We also detected a mutation out of the QRDR but within the gene *gyrB* in a quinolone resistant *M. bovis* isolate 869 (Arg58Gly), although two other mutations in the QRDR were also associated (Table 3).

## 4. Discussion

The analysis of a set of strains collected in Spain during 2023 has shown, for the first time, the circulation of *M. bovis* biotype ST-1 in the country. Previous data available about the strains analyzed since 2016 only revealed the presence of two biotypes circulating in Spain (ST-2 and ST-3). Despite the limitations of the sampling, the data obtained also suggest that, like the others, this biotype is also endemic in the country.

The presence of the ST-1 subtype should not be considered surprising, as the movement of animals in and out of Spain is very frequent, although it is interesting that the characterization of more than 100 isolates carried out between 2016 and 2021 did not previously identify its presence. The ST-1 subtype, to which the reference strain PG-45 belongs, was described as receding since the year 2000 [7,11]. This loss of importance during these decades was associated to its higher susceptibility to antimicrobials compared to the ST2 and ST3 biotypes [18,19]. However, subsequent data showed that other factors must be involved. For example, in France, 80% of isolates are ST-2, whose strains are sensitive to fluoroquinolones compared to ST-3, which curiously represents the predominant clone in Spain [5]. Probably, unknown factors other than antimicrobial resistance must be involved in the evolutionary advantages of the different subtypes. In this sense, recent data showed that the French ST-2 population is composed of two lineages that emerged from independent introductions of international strains, emerging the first one around 2000 supplanting the previously established subtype 1 [4].

Regarding the antimicrobial susceptibility of ST-1 strains circulating in Spain, the results show, as in ST-2 and ST-3, their multiresistance to macrolides or lincosamides, confirming previous results [18,20,21]. In this sense, *M. bovis* can retain resistance for macrolids even when antimicrobial selection pressure is removed [21]. Different phenotypic subgroups could be established in relation to second-generation FQN; sensitive (such as most of the ST-2 isolates), resistant (such as most of the ST-3 isolates) or even an intermedium level of resistence. Although it should be analyzed with caution, given the small number of Spanish ST-1 isolates analyzed, the higher antimicrobial resistance of these isolates compared to previous data, could explain the apparent re-emergence of this biotype in countries such as France, where, since 2014, ST-1 isolates have started to be collected again [7]. As previously observed by other authors, valnemulin was the only antimicrobial tested that was demonstrated activity against ST-1, similarly to its efficacy against ST-2 and ST-3 biotypes, [5,22]. This efficacy is probably caused by an antimicrobial without official use for bovines, at least in Spain.

Related to the resistance to FQN, the analysis of the QRDR region of ST-1 strains circulating in Spain has shown the presence of mutations that could be related to resistence in some of them, but also in other areas of the genes analyzed. Previous studies evidenced that with the same number of in vitro passages, ST-1 biotypes failed to acquire substitutions in these targets [7], although the presence of these mutations could be the result of the pressure exerted by the use of fluoroquinolones or even the presence of new, unanalyzed resistance mechanisms acquired through various mechanisms such as horizontal transfer or others, whose presence has recently been demonstrated in vivo in Spanish strains [23].

From a practical point of view, in order to establish effective antimicrobials treatments in affected farms, the best method is MIC. Sensitivity to antimicrobials can be conducted in vitro but at the moment, it is complex to perform a MIC test for each animal presenting respiratory symptomatology, even when the presence of *M. bovis* is suspected, due to the time required for *M. bovis* isolation or the MIC technique. On the other hand, *M. bovis* is a complex microorganism with several mechanisms involved in variability as the presence of ICEs, IS or other strategies oriented to chronify in the host. Several techniques have been developed demonstrating its high grade of genomic variability such as MLST, obtaining a greater discrimination. Despite that, the presence of different profiles of sensitivity to quinolones that could be associated to the majority of the strains grouped in a *polC* subtype, an easiest and fastest technique that could be adapted as a diagnostic method, opened the way of orienting the treatments according to the subtype present in the farms. However, the presence of ST-1 subtype isolates in Spain, showing a variable sensitivity to quinolones, could limit the possibilities of putting this strategy into practice, because, in effect, unlike ST-2 and ST-3, molecular characterization does not allow to predict some sensitivity to FQN of most of the isolates, which will require the corresponding MIC, with a consequent loss of time, essential for the early treatment of mycoplasmas.

In the same line, the analysis of a *M. bovis* ST-1 isolates recovered from different animals (*n* = 8) affected with respiratory symptoms and belonging to the same feedlot, evidenced the variability of MIC profiles among the different isolates, suggesting the presence and circulation of different clones. As in other cases of chronic respiratory disease where other biotypes have been implicated, the possibility of co-infections between biotypes in the same flock and feedlot is also possible with ST-1 Unfortunately, this variability further limits the therapeutic possibilities of establishing targeted treatments in the event of an increase in the number of mycoplasma-infected animals, although it is also no guarantee of effective treatment of animals with respiratory symptoms [5,7].

In conclusion, our study revealed the extended circulation of a third *polC* ST-biotype of *M. bovis* in Spain. Circulating isolates are now divided into three groups, ST-1 to ST-3, all being resistant to macrolides, lincosamides. Most ST-3 isolates circulating in Spain are resistant to FLQ, while ST-2 remained resistant and ST-1 sensibility seem to be variable.

## Figures and Tables

**Table 1:** Epidemiological information available of the sampled animals in which *Mycoplasma bovis* subtype ST-1 was detected.

Isolates	Animal	Age	Area	Symptoms
991	Calf	6–8 months	Murcia	Pneumonia
100EX	Calf	6–8 months	Aragón	Pneumonia
103EX	Calf	6–8 months	Castile and León	Pneumonia
98EX	Calf	6–8 months	Castile La -Mancha	Pneumonia
91EX	Calf	1 month	Catalunya	Pneumonia
908	Calf	6–8 months	Murcia	Pneumonia
916	Calf	6–8 months	Murcia	Pneumonia
913	Calf	6–8 months	Murcia	Pneumonia
914	Calf	6–8 months	Murcia	Pneumonia
923	Calf	6–8 months	Murcia	Pneumonia
915	Calf	6–8 months	Murcia	Pneumonia
928	Calf	6–8 months	Murcia	Pneumonia
930	Calf	6–8 months	Murcia	Pneumonia
99EX	Calf	6–8 months	Murcia	Pneumonia
869	Calf	6–8 months	Murcia	Pneumonia
945	Calf	6–8 months	Murcia	Pneumonia

**Table 2:** MIC ranges, MIC_50_ and MIC_90_ of ST-1 Spanish *M. bovis* isolates.

		Macrolides					FLQ				TET	
MIC Parameter	Tul	Gam	Eri	Tyl	Spy	Lin	Enr	Marb	Dan	Dox	Oxy	Val
MIC Range	2–>16	>16	>16	8–>16	0.25–>16	0.5–>16	0.0625–>16	0.25–>16	0.125–8	0.5–8	2–>16	0.0312
MIC_50_	>16	>16	>16	>16	>16	>16	0.25	0.5	0.25	1	4	0.0312
MIC_90_	>16	>16	>16	>16	>16	>16	0.5	1	0.5	4	>16	0.0312

MIC values are given in µg/mL. Tul = Tulathromycin; Gam = Gamithromycin; Eri = Eritromycin; Tyl = Tylosin; Spy = Spyramycin Lin = Lincomycin; Enr = Enrofloxacin; Marb = Marbofloxacin; Dan = Danofloxacin; Dox = Doxycycline; Oxy = Oxytetracycline; Val = Valnemulin.

**Table 3:** List of point mutations in the *gyrA*, *gyrB*, *parE* and *parC* QRDR identified in *M. bovis* isolates and associated MIC values for FLQ.

Isolate	*gyrA*	*gyrB*	*parE*	*parC*	MIC (µg/mL) ^a^		
	83 ^b^	105	109	278	Enr	Marb	Dan
**PG45**	Ser	Asp	Gln	Ser	0.125	0.5	0.125
991	-	-	-	-	2	0.5	1
100EX	-	-	-	-	<0.063	0.5	0.5
103EX	-	-	-	-	<0.063	1	0.25
98EX	-	-	-	-	<0.063	0.25	0.125
91EX	-	-	-	-	<0.063	0.5	0.25
908	-	-	-	-	0.25	0.5	0.125
916	-	-	-	-	0.25	0.5	8
913	-	-	-	-	<0.063	0.25	0.125
914	-	-	-	-	<0.063	0.25	0.125
923	-	-	-	-	8	+8	4
915	-	-	-	-	<0.063	0.5	0.125
928	-	-	-	-	8	+8	4
930	-	-	-	-	8	+8	4
99EX	-	-	-	-	0.25	0.5	0.125
869	Phe	Asn	-	Ile	8	+8	4
945	-	-	Asn	-	+8	+8	4

Amino acid numbering refers to positions in *Escherichia coli* K12. ^a^ []. ^a^ Enr = Enrofloxacin; Marb = Marbofloxacin; Dan = Danofloxacin. ^b^ Mutations associated with FLQ resistance in previous studies.

## Data Availability

The original contributions presented in the study are included in the article.

## Appendix A

Preparation of the modified medium SP4 [12]. The modified medium is composed of three parts (A, B and C). Part A is composed of 4.2 g of Difco PPLO broth (BD), 6.4 g of Bacto Peptone (BD), 12 g of Bacto Tryptone (BD) and 724 mL of deionized water. The solid medium includes 7 g of European Bacteriological Agar (Conda-Pronadisa, Madrid, Spain). The pH is adjusted to 7.8 and then part A is autoclaved at 121 °C for 20 min. Part B is composed of 60 mL of RPMI-1640 (Sigma-Aldrich), 21 mL of fresh yeast extract 50% *w*/*v*, 2.4 g of yeast extract (Conda-Pronadisa), 4.8 mL of phenol red 0.5%, (Sigma-Aldrich) and 0.642 g of ampicillin sodium salt (Fisher bioreagents). The pH is adjusted to 7.2 and then part B is filter sterilized through a 0.2 µL pore size filter. Part C is composed of 251 mL of heat-inactivated horse serum (Hyclone) for 30 min at 56 °C.
